# Supplementary material for: The causal relationship between the human gut microbiota and pyogenic arthritis: a Mendelian randomization study
Source: Front Cell Infect Microbiol. 2024 Nov 26;14:1452480. doi: 10.3389/fcimb.2024.1452480 (PMC11629706; doi:10.3389/fcimb.2024.1452480)
Supplement: Supplementary file 1 [file DataSheet1.zip › mendelian test/ebi-a-GCST90027855.csv_leave_one_out.pdf]

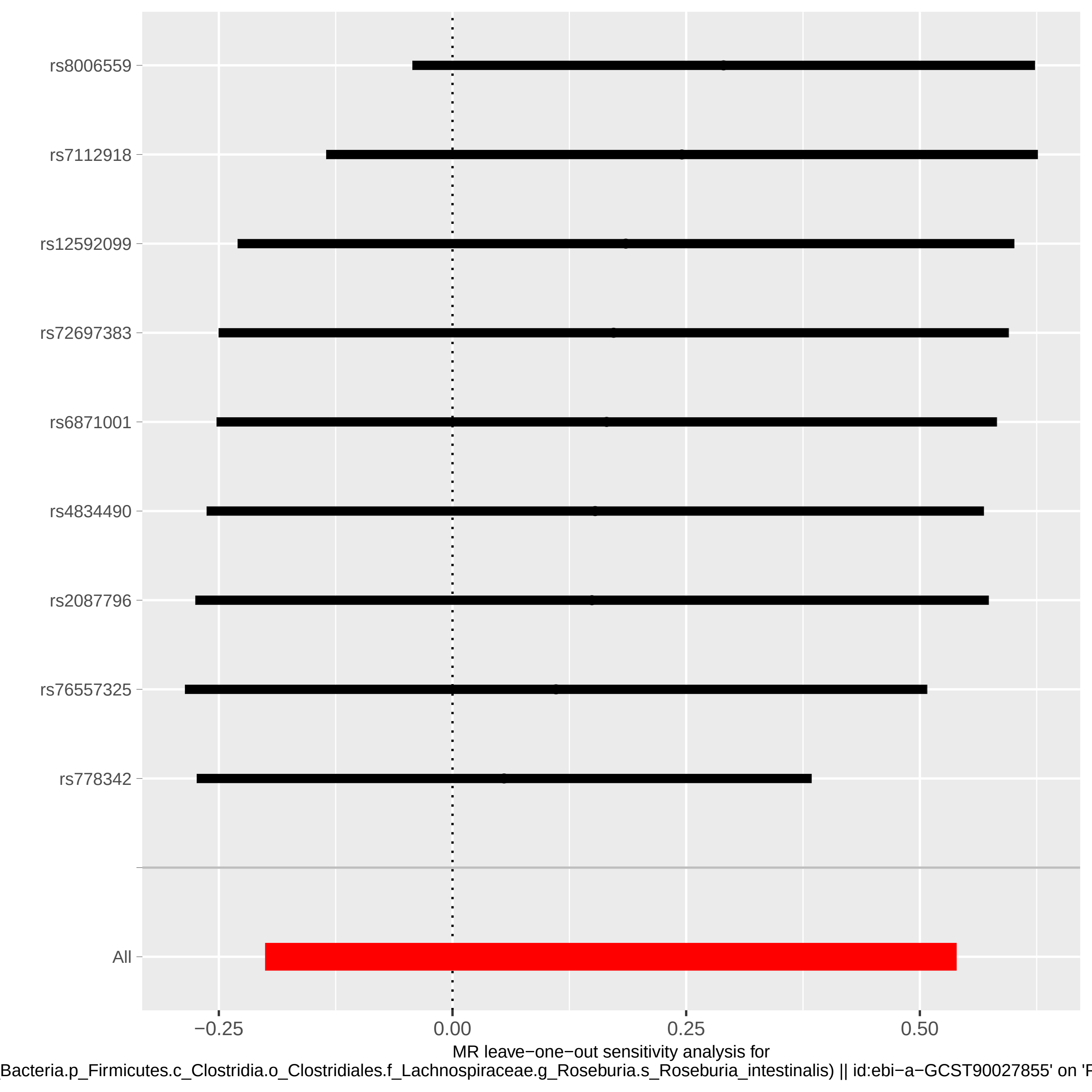
rs8006559
rs7112918
rs12592099
rs72697383
rs6871001
rs4834490
rs2087796
rs76557325
rs778342
All
−0.25
0.00
0.25
0.50
MR leave−one−out sensitivity analysis for
Bacteria.p_Firmicutes.c_Clostridia.o_Clostridiales.f_Lachnospiraceae.g_Roseburia.s_Roseburia_intestinalis) || id:ebi−a−GCST90027855' on 'F
